# Increased expression of EZH2 indicates aggressive potential of urothelial carcinoma of the bladder in a Chinese population

**DOI:** 10.1038/s41598-018-36164-y

**Published:** 2018-12-12

**Authors:** Xiaozhou Zhou, Nan Liu, Jingqi Zhang, Huixiang Ji, Yuting Liu, Jin Yang, Zhiwen Chen

**Affiliations:** 1 Urology Institute of People Liberation Army, Southwest Hospital, Third Military Medical University (Army Medical University), Chongqing, China; 2 Chongqing University Cancer Hospital & Chongqing Cancer Institute & Chongqing Cancer Hospital, Chongqing, China; 3 Department of Cell Biology, The Third Military Medical University (Army Medical University), Chongqing, China

## Abstract

Here, we attempt to better define the long-term outcomes of radical cystectomy (RC) for urothelial carcinoma (UC) in a Chinese population and to investigate the relationship between EZH2 protein expression levels and the clinicopathological parameters and outcomes in patients with UC. We detected the relative EZH2 protein expression levels by immunohistochemistry in tumour specimens from a cohort of 189 Chinese UC patients. In patients who underwent RC, the 5-year cancer-specific survival (CSS) and overall survival (OS) were 69% and 61% respectively. EZH2 expression was increased in UC compared with normal urothelium. The expression levels of EZH2 were elevated in parallel with tumour stage (p = 0.001) and tumour grade (p = 0.001) and were increased in cases with lymph node metastasis compared with node-negative cases (p = 0.018). Kaplan-Meier analyses showed that higher EZH2 expression was related to significantly shorter CSS and OS in patients who underwent RC. High EZH2 expression was associated with worse CSS (HR = 3.51; p = 0.037) and OS (HR = 2.15; p = 0.047) in the univariate analysis, but only lymph node invasion maintained its predictive value for CSS in a multivariate model. This contemporary and homogeneous single-centre series found acceptable outcomes for Chinese UC patients who underwent RC. Clinically, our retrospective studies suggest that EZH2 levels can be used to identify more aggressive phenotypes in UC patients, thereby improving our prognostic knowledge.

## Introduction

Bladder cancer is the ninth most common malignancy and an important cause of mortality; in 2012 alone, an estimated 430,000 new cases and 165,000 deaths occurred worldwide, with 75% of the burden in men^1^. The most common histological type of bladder cancer is urothelial carcinoma (UC), which accounts for >95% of all primary bladder malignancies combined^2^. To prolong the length and quality of patients’ lives, the clinical spectrum of bladder cancer was divided into variable categories that differ in prognosis, management and therapeutic goals. Although non-muscle-invasive bladder cancer (NMIBC) is considered a non-invasive tumour, the risk of recurrence is as high as 78%, and the risk of progression is as high as 45%, which leads to cancer mortality in 16–23% of patients after conservative bladder-sparing treatment within 5 years^3^. Moreover, during the routine follow-up of patients with muscle-invasive bladder cancer (MIBC) after surgery, more than half of all metastases are found after the appearance of symptoms, and the overall survival is 66% at 5 years^4^.

The survival rate of patients with bladder cancer varies among different ethnic groups, genders and age groups^5^. David Yee *et al*. demonstrated poorer survival in blacks compared with whites, and the cancer-specific survival was similar for whites, Hispanics, and Asian/Pacific Islanders with a median follow-up of 4.2 years^6^. Moreover, considering the patients with urothelial carcinoma, the African-Americans appear to be independently associated with a higher risk of tumour recurrence after radical cystectomy (RC)^7^. However, research on bladder cancer outcomes in Chinese populations is limited.

Enhancer of zest homolog 2 (EZH2) serves as a catalytic subunit in the polycomb repressive complex 2 (PRC2), which methylate lysine27 of histone H3 (H3K27) to promote the transcriptional silencing of many genes^8^. Increasing study have proposed that overexpression of the EZH2 gene occurs in various human malignance such as prostate cancer, breast cancer, colorectal cancer and lung cancer^9–15^. Furthermore, EZH2 overexpression also contributes to cancer development and progression via chromatin modifications, which occur as a result of the epigenetic activation of carcinogenic signalling cascades and the silencing of tumour suppressor genes; it has also played a role in cell proliferation, differentiation, invasion, and metastasis^16^. Thus, EZH2 has oncogenic properties. An increasing amount of meta-analysis data has shown that the abnormal expression of EZH2 is a prognostic factor for patients with various human cancers such as lung cancer, breast cancer and digestive cancers^10,13,15^. Previous studies have shown that the overexpression of EZH2 might serve as a marker of a poor prognosis. Chen *et al*. even showed that EZH2 might be an independent prognostic factor for multiple survival assessments in different cancers^17^.

Although several previous studies have proposed that EZH2 may be a potential marker for bladder cancer^18–22^, the results regarding the relationship between EZH2 expression and tumour prognosis are inconclusive, and no consensus has been reached. As far as we know, all the published studies have confirmed that the expression levels of EZH2 protein were increased in tumours compared with normal urothelium. However, the association of EZH2 expression levels with the biological behaviour of tumours is contradictory. Moreover, Hinz *et al*.^18^reported that patients with UC and EZH2 overexpression exhibited a worse prognosis including a shorter recurrence-free survival and overall survival. In contrast, a study by Warrick *et al*.^22^ in 20I6 suggested that EZH2 expression in bladder cancer was not a predictor of oncologic outcome.

The expression status of EZH2 in Chinese patients with UC has not yet been reported. Therefore, one objective of this study was to assess whether patients undergoing RC for UC in China may exhibit outcomes similar to those of whites and Hispanics. We further investigated the difference in EZH2 levels between tumours and benign tissues in Chinese patients with UC and explored whether elevated EZH2 expression could be a valuable biological marker associated with aggressive and invasive potential of UC of the bladder.

## Results

### Clinicopathological characteristics

The present study cohort consisted of 189 patients, 153 of whom underwent RC and 36 of whom underwent transurethral bladder cancer resection. The cohort consisted of 34 females (18.0%) and 155 males (82.0%) with a median (range) age of 65 (24–86) years. Two patients had pTis tumours, 21 patients had pTa, 41 patients had pT1, 97 patients had pT2, and 28 patients had >pT2 tumours. One hundred and fifty patients had low-grade tumours, and 39 had high-grade tumours. The detailed clinicopathologic characteristics of the 189 patients are presented in Table 1. Considering the 153 RC patients alone, most patients (88.9%) had bilateral pelvic lymph node dissection with a node-positive rate of 16.2%. None of the patients received neoadjuvant chemotherapy. In all, 233 tissue specimens including 44 specimens of adjacent benign urothelium (Table 1) from the whole patient cohort were evaluated for EZH2 expression.

**Table 1:** Clinicopathologic characteristics of patient cohort with urothelial carcinoma.

Patients (n)	189	44^*^
Age (year)		
Median (range)	65.0 (26–84)	65.5(26–84)
Mean ± SD	63.9 ± 11.3	63.6 ± 12.5
Sex		
Male n (%)	155(82.0)	38(86.4)
Female n (%)	34 (18.0)	6(13.6)
Smoking history		
Smoker	79 (41.8)	18(40.9)
Nonsmoker	110 (58.2)	26(59.1)
Tumor grade n (%)		
Low grade	150 (79.4)	35(79.5)
High grade	39 (20.6)	9(20.5)
Pathologic stage n (%)		
pTa	21 (11.1)	2(4.5)
pTis	2 (1.1)	1(2.3)
pT1	41 (21.7)	6(13.6)
pT2	97 (51.3)	25(56.8)
pT3	15 (7.9)	4(9.1)
pT4	13 (6.9)	6(13.6)
Operative type n (%)		
TUR	36 (19.0)	2(4.5)
RC + Neobladder	105 (55.6)	24(54.5)
RC + ileal conduit	48 (25.4)	18(40.9)
Pathologic nodal stage n (%)^#^		
pN unknow	17(11.1)	7(16.7)
pN negative	114 (83.8^$^)	30(85.7^$^)
pN positive	22 (16.2^$^)	5(14.3^$^)
Follow up (months)^#^		
Median (range)	68.0 (0–129)	62.0(3–126)
Mean ± SD	62.5 ± 41.7	58.1 ± 39.5
Survival situation (%)^#^		
Dead	68 (42.8)	18(42.9)
Living	65 (40.9)	17(40.5)
Lost	26 (16.4)	7(16.7)

TUR: Transurethral bladder cancer resection; RC: radical cystectomy;

^*^The part of the patients who provided the specimens of adjacent benign urothelium.

^#^Considering patients undergoing radical cystectomy only.

^$^Calculate the proportion in patients receiving lymphadenectomy.

### Increased EZH2 expression is associated with aggressive biological behaviour in bladder cancer

**Figure 1:**
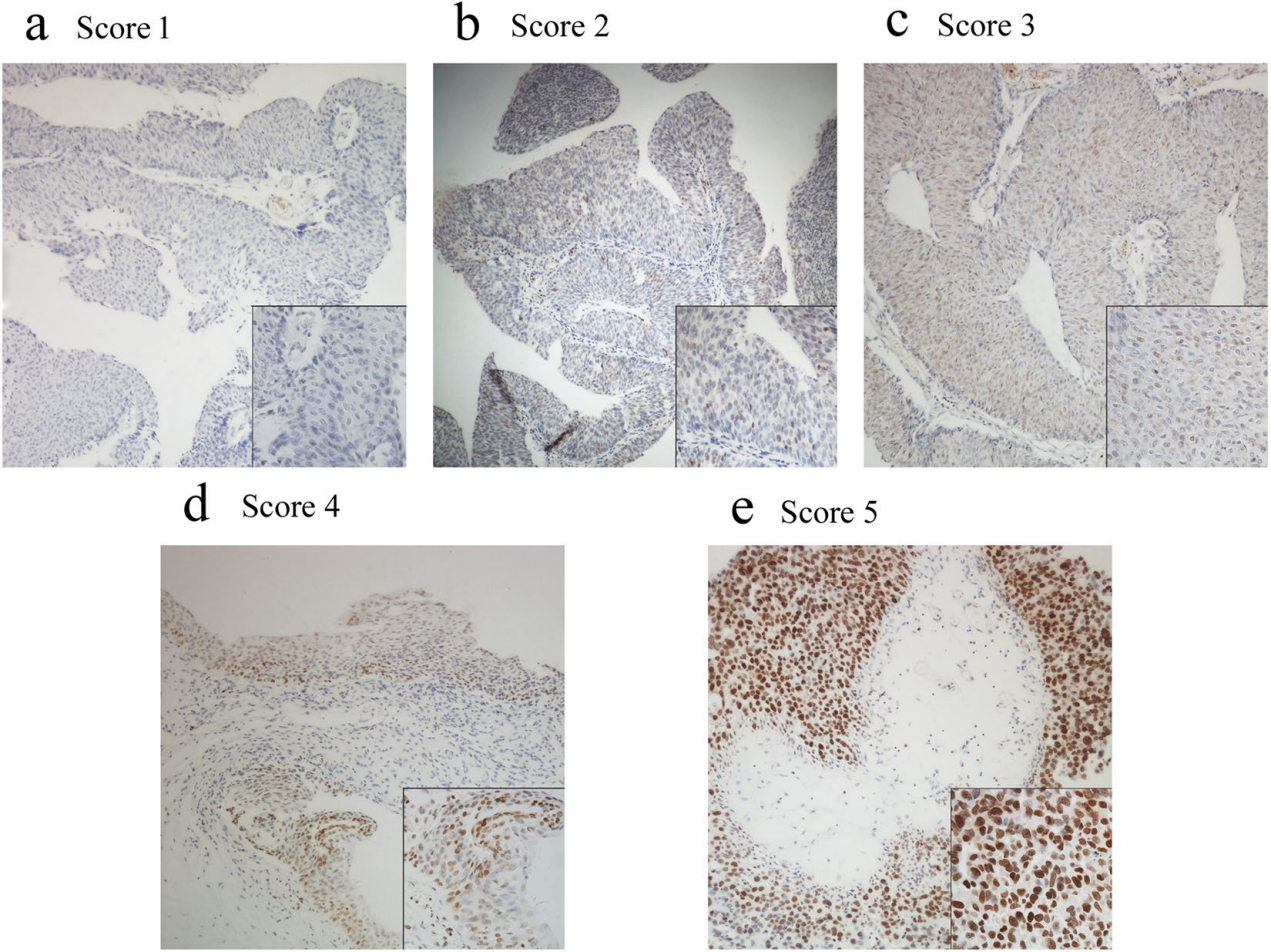
Representation images of immunohistochemical staining intensities of EZH2 protein in urothelial carcinoma tissues. EZH2 immunoreactivity was found in the nucleus of tumor cells. EZH2 expression was classified into 1 to 5 categories according to the EZH2 staining intensity and extension values. (**a**) Score = 1 (**b**) Score = 2 (**c**) Score = 3 (**d**) Score = 4 (**e**) Score = 5. Magnification, ×100 (**a**–**e**) and for lower right-hand corner of (**a**–**e**), ×400.

Immunohistochemical staining showed that EZH2 protein was mainly expressed in the nuclei of urothelial cells. Representative images of EZH2 expression by immunohistochemistry are shown in Fig. 1.

The final immunostaining score of the tumour specimens were group 1 in 4 patients, group 2 in 20 patients, group 3 in 20 patients, group 4 in 60 patients and group 5 in 85 patients. Considering the specimens of adjacent benign urothelium were group 1 in 26 patients, group 2 in 14 patients, group 3 in 2 patients, group 4 in 1 patient and group 5 in 1 patient. Regarding EZH2 as a binary variable, high EZH2 expression was seen in 76.7% of tumours and in 4.5% of benign urothelium. EZH2 expression was detected at significantly higher levels in neoplastic tissue compared with benign urothelium (Wilcoxon signed rank test, p < 0.001). Comparisons of EZH2 expression among different clinicopathologic characteristics of the tumours are shown in Table 2. Overall, EZH2 expression levels were elevated in parallel with tumour stage (Kruskal-Wallis test p = 0.001) and were also increased in MIBC compared with NMIBC according to the Mann-Whitney test (p = 0.027). High-grade tumours exhibited increased staining compared with low-grade tumours (Mann-Whitney U-Test, p = 0.001). Moreover, considering the lymph node status of patients who underwent RC, the rate of lymph node metastasis was associated with significantly increased EZH2 staining compared with non-lymph node metastasis (Mann-Whitney test, p = 0.018). No difference was observed in EZH2 protein expression among the patient subgroups, which were divided according to sex (p = 0.628), age (p = 0.962) and smoking history (p = 0.832).

**Table 2:** Comparisons of EZH2 expression among different clinicopathologic characteristics of urothelial carcinoma following surgical treatment.

	EZH2 high n (%)	EZH2 low n (%)	^*^p
Sex			
Male	120 (77.4)	35 (22.6)	0.628
Female	25 (73.5)	9 (26.5)	
Age			
>60 year	95 (76.6)	29 (23.4)	0.962
≤60 year	50 (76.9)	15 (23.1)	
Smoking history			
Smoker	60 (75.9)	19 (24.1)	0.832
Nonsmoker	85 (77.3)	25 (22.7)	
Tumor grade			
Low grade	107 (71.3)	43 (28.7)	0.001
High grade	38 (97.4)	1 (2.6)	
Pathologic stage			
MIBC	102 (81.6)	23 (18.4)	0.027
NMIBC	43 (67.2)	21 (32.8)	
Lymph nodal status			
Positive	22 (100.0)	0 (0.0)	0.018
Negative	90 (78.9)	24 (21.1)	

MIBC: muscle invasive bladder cancer; NMIBC: non-muscle invasive bladder cancer

* Mann–Whitney test.

### Survival analysis of patients undergoing RC

**Figure 2:**
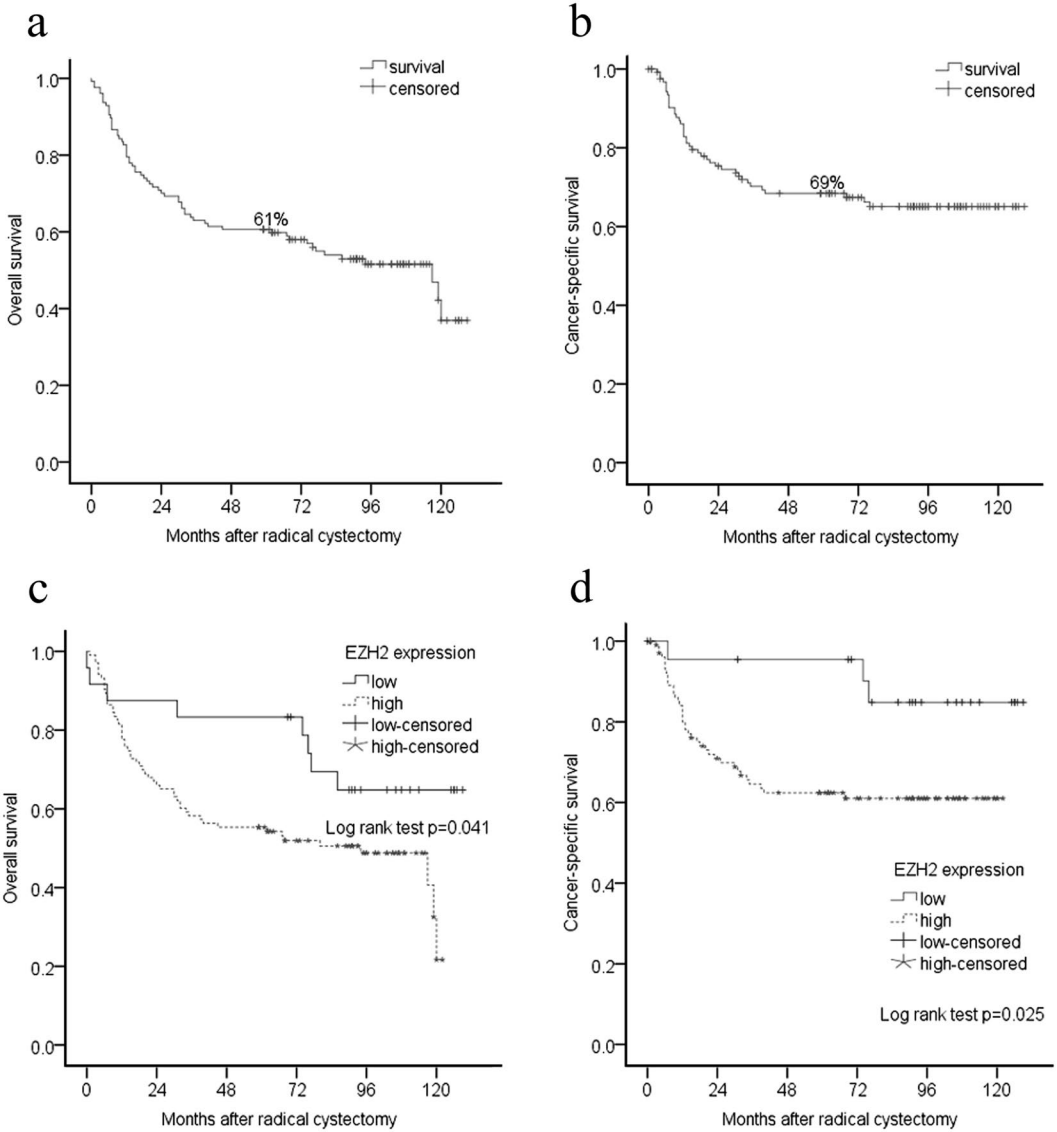
Kaplan-Meier analysis of survival rate for radical cystectomy population. (**a**) Overall survival (OS) rate for the series of 153 patients. Five-year OS was 61%. (**b**) Cancer specific survival (CSS) rate for the series. Five-year CSS was 69%. (**c**) OS stratified according to EZH2 protein expression. OS was significantly longer for patients with low EZH2 expression compared to patients with EZH2 expression high (Log rank test p = 0.041). (**d**) CSS stratified according to EZH2 protein expression. CSS rate showed significant differences between low and high EZH2 expression (p = 0.025).

The baseline clinical and pathological characteristics of patients who underwent RC are shown in Table 1. Follow-up was defined as the interval from RC until death or until December 2017. The data of 127 patients (83.0%) were available with a median follow-up time of 68.0 months (range 0–129 months) following RC. Of the 127 patients, 62 patients had died, including 41 patients who had died from cancer recurrence and metastasis. The 30-day mortality rate in this series was 2.0%. A total of 3 patients died within 30 days after cystectomy, and these deaths were related to surgical complications. The 5-year OS and CSS rates for the RC population were 61% and 69%, respectively (Fig. 2a,b). Increasing age (hazard ratio (HR) = 1.04, CI 95% 1.01–1.08 p = 0.012) and lymph node-positive (HR = 3.64, CI 95% 1.71–7.75, p = 0.001) disease were associated with significantly worse OS in a multivariate Cox regression analysis. Moreover, only lymph node-positive disease predicted worse CSS (HR = 3.62, CI 95% 1.52–8.60, p = 0.004).

### EZH2 expression is associated with oncologic outcomes but does not provide independent prognostic information

We further explored the potential predictive value of EZH2 protein expression in UC patients who underwent RC (n = 153). To assess the relationship between EZH2 expression and clinical outcomes, we performed a Kaplan-Meier analysis to determine whether EZH2 protein expression status were related to CSS and OS following RC. The analyses showed that CSS (p = 0.025) and OS (p = 0.041) were significantly shorter in patients with high expression of EZH2 compared with those with low expression of EZH2. Figure 2c,d depict the differences in CSS and OS according to the EZH2 expression status.

In exploratory analyses, univariate and multivariate Cox regression models were applied to further assess whether EZH2 protein expression levels were correlated with CSS and OS. Compared with patients with low expression of EZH2, the hazard ratios of overall death and cancer-related death for those with high EZH2 expression were 2.15 (range 1.01–4.56) (p = 0.047) and 3.51 (range 1.08–11.38) (p = 0.037), respectively, in the univariate models. Moreover, we performed multivariate Cox regression analyses to evaluate whether EZH2 may provide independent prognostic value in our cohort. In the multivariate model, no significant correlation was presented between EZH2 protein expression and OS (p = 0.577) or CSS (p = 0.201). Lymph node invasion maintained its predictive value in both cancer-related death (HR = 3.23, CI 95% 1.43–7.32, p = 0.005) and overall death (HR = 3.28, CI 95% 1.60–6.71, p = 0.001). The Bootstrapping resampling tests showed robust conclusions (See Supplementary Data Files S1).

## Discussion

In the present study, RC performed at our institution provides acceptable clinical outcomes (5-year OS and CSS rates were 61% and 69%, respectively) and low postoperative mortality. As previously reported, lymph node status is a key prognostic factor for patients undergoing RC for bladder cancer^23^. We did not find that the pathologic stage is an independent survival predictor in this cohort, presumably due to the great proportion of pT2 patients. Moreover, considering that or study included more organ-confined and lymph node-negative UC, the patient cohort should have better outcomes compared with what is described in other recent reports^4,23,24^. The possible reason for this is that the included patients who underwent RC did not receive neoadjuvant chemotherapy, and a portion of them did not even undergo bilateral pelvic lymph node dissection. Moreover, loss to follow-up after RC may decrease the survival benefit. Nevertheless, this contemporary and homogeneous single-centre series supports the idea that Chinese populations undergoing RC for urothelial carcinoma in China have outcomes similar to those of whites and Hispanics.

As far as we know, the conventional prognostic evaluation of bladder cancer is mainly according to tumour staging and histological grading^25^. However, there is increasing evidence that the cellular and molecular characteristics of primary tumour contribute to the great differences of prognosis of bladder cancer even with the same tumour stage and/or grade^25–27^. Advances in genomics have significantly improved our understanding of UC development and illuminated various driver genetic pathways and changes. The integration of genomics and proteomics lays a foundation for genomic-based classification of bladder cancer based on endogenous molecular subtypes. Robertson *et al*.^25^ analysed 412 cases of MIBC characterized by multiple analytical platforms of TCGA (The Cancer Genome Atlas). They identified subgroups with different biological characteristics and clinical outcomes by clustering mRNA, long non-coding RNA, and microRNA expression. A high-mutation subset with 75% 5-year survival was identified by mutation clustering. Meanwhile, their analyses identified five expression subtypes that can be used to stratify patients and inform clinical trials. Hedegaard *et al*.^27^ carried out multiple platforms analysis of NMIBC and suggested two main categories (basal-like and luminal-like) according to different clinical and biological characteristics. Therefore, it is urgent to identify new and reliable markers to predict the outcomes of patients effectively and accurately.

Increasing evidence that links EZH2 activity to cancer has recognized EZH2 as an improtant driver of cancer initiation, growth and progression. Specifically, a recent genetic mouse model showed that interception of an Rb-E2F-EZH2 signalling pathway caused UC^28^. Whole transcriptional analyses of mouse and human bladder tumours showed that EZH2 had significant overlap in function, and confirmed that EZH2 played a critical role in the down-regulation of gene expression programs. Furthermore, tumour progression in humans with NMIBC is associated with increased E2F and EZH2 expression and EZH2 mediated gene expression inhibition. These findings indicated that the increased expression of EZH2 is an important event that reveals epigenetic modifications in bladder cancer. Our findings agreed with data from several earlier preliminary studies^18–22^ and definitely demonstrated that EZH2 protein was constitutively highly expressed in UC tissues.

Failure to identify low-risk subgroups of cancers can often lead to overtreatment of cancer patients. Biomarkers of prognosis may help to enhance the ability to predict whether a patient’s cancer is going to recur after surgery. Through an immunohistochemical analysis of a cystectomy series involving 301 patients, Warrick *et al*.^22^ recently showed that aberrant higher EZH2 expression was not associated with more advanced bladder cancer. However, in the present study, our results suggested that increased expression of EZH2 was significantly correlated with more aggressive biological behaviour including higher grade, stage and lymph node metastasis. These data are consistent with some published results^18,21^ which demonstrated that the high expression of EZH2 was positively correlated with the increase of aggressive behaviour, which indicates that the expression EZH2 protein may be involved in the progression of UC through its function as an oncogene. In addition, no difference was observed in EZH2 protein expression among the patient subgroups that were divided according to gender, age and smoking history. Numerous studies have suggested that EZH2 might be a meaningful prognostic factor for multiple survival assessments in a variety of cancers. In some cancers, such as human breast cancer, lung cancer and digestive cancers^10,13,15^, the overexpression of EZH2 proteins have been associated with the poor prognosis of the malignancies. In our study, the univariate analysis showed that higher EZH2 expression was associated with worse outcomes of patients with UC, which suggests that EZH2 may play an important role in the progression of UC. However, in the multivariate analysis, which controlled the effect of tumour lymph node staging, the expression levels of EZH2 no longer predicted the prognosis of patients. This phenomenon indicated that the higher expression of EZH2 was dependent on aggressive pathological hallmarks including grade and stage in human bladder cancer. Analogous results^18^ have been reported in a previous study that showed that high expression of EZH2 were positively correlated with tumour-related death by quantitative RT-PCR in tumour specimens from a cohort of 100 patients.

Based on the finding that EZH2 confers stemness and regulates differentiation during embryonic development^29^, EZH2 might also be involved in the regulation of stem cells in human bladder cancer. Moreover, EZH2 can induce epithelial mesenchymal transition (EMT) and increase the metastatic potential of prostate cancer cells by downregulating DAB2IP, a tumour suppressive Ras GTPase-activating protein (RasGAP)^30^. Further, EZH2 promotes EMT by epigenetically suppressing E-cadherin via canonical H3K27me3 modification of its promoter^31–33^, facilitated by MEK/ERK signalling^34^. Consequently, the inhibition of E-cadherin is associated with poor clinical outcomes in advanced malignancies^35^. Recent researches^36^ also showed that abnormal expression of EZH2 in cancer cells can assist them to regulate immune response and immunotherapy. Moreover, EZH2 plays a key role in T and NK cells mediated immune evasion^37^. These results suggest that EZH2 may act as a useful target for the treatment of bladder cancer. It was reported that a selective chemical inhibitor of EZH2 could block H3K27 methylation and kill mutant lymphoma cells^38^, which suggests that cancer may be treated by targeting EZH2 as a therapeutic strategy. Indeed, early results with the EZH2 inhibitor EPZ6438 in patients with B-cell lymphomas and advanced solid tumours (NCT01897571) are encouraging.

Focusing on metastasis-driving gene expression pathways, Edwin Wang *et al*.^39,40^ had developed a prediction model that identified prognostic markers using tumour gene microarrays. They showed that those genes that were related to the survival of patients were more likely to be related to metastasis. However, the robustness of gene signatures is significantly affected by interpatient and intratumor heterogeneity which blocks the identification of reliable cancer biomarkers. Bootstrapping is one of the common resampling tests for evaluating the estimated sample distribution by replacing the original sample with other sample, most often with the purpose of deriving reliable estimates of standard errors and confidence intervals of a population parameter like a median, mean, proportion, odds ratio, regression coefficient or correlation coefficient. Although the resampling tests showed our conclusions were robustness. Considering the limited sample size of each study including ours, the heterogeneity may lead to the contradictory results of the predictive value of EZH2. Recently, Shu-Ping Wang *et al*.^41^ showed that p53 as a tumour suppressor also suppresses cancer invasion and metastasis. Thus, the mediators of metastasis may also have dual functions, providing local advantages for malignant progression of primary tumour and distal advantages for metastasis. EZH2 is now widely regarded as an oncogene. Whether it has both functions in bladder cancer requires further validation in a larger cohort. Clinically, our retrospective studies suggest that EZH2 levels can be used to identify more aggressive phenotypes in UC patients, thereby improving our prognostic knowledge.

In summary, to our knowledge, this is the first study to date that has evaluated EZH2 expression in bladder cancer in a Chinese population. This contemporary and homogeneous single-centre series found acceptable outcomes for Chinese patients with UC who underwent RC. Elevated EZH2 expression in UC is strongly associated with aggressive tumour biological behaviour and poor prognosis but does not provide independent prognostic information in this Chinese population. This study has a practical therapeutic implication for the selection of appropriate methods based on different molecular defects, including a conserved or aggressive surgical approach,. It is worthwhile to further clarify the role of EZH2 in the initiation and progression of UC and to investigate its predictive value of clinical outcomes in a larger UC patient cohort.

## Methods

### Patient specimens and clinical data

Bladder cancer samples were prospectively collected from 189 patients who were treated for UC of the urinary bladder at Southwest Hospital, The Third Military Medical University. One set of specimens was procured from 153 patients at the time of RC by one surgeon (Chen Zhiwen) between 2007and 2012, and the other set was obtained from 36 patients at the time of transurethral resection in 2012. In 44 patients, nontumor samples were also obtained from grossly uninvolved adjacent bladder urothelium that was at least 2 centimetres away from the visible tumour. Patients who had previously received neoadjuvant chemotherapy were excluded from the study. All selected tissues were immediately fixed in a 10% formalin solution. The case history and pathologic data of the cohort were retrospectively reviewed. The Institutional Review Board of Southwest Hospital, The Third Military Medical University approved the tissue procurement and data collection in accordance with the relevant guidelines and regulations (The WMA Declaration of Helsinki and the Department of Health and Human Services Belmont Report). All patients provided informed consent when indicated by the Institutional Review Board. Patient and tumour demographics are listed in Table 1.

Tumour classification and staging were based on the final reports of the tissues submitted to the Department of Pathology and were performed according to the American Joint Committee on Cancer (AJCC) TNM Staging System. Histopathologic grading was performed according to the 2004 WHO/ISUP version^42^.

### Immunohistochemistry

Five-micrometre-thick tissue sections were cut from the formalin-fixed, paraffin-embedded tissue specimens and were deparaffinized in xylene followed by rehydration in a graded series of ethanol. Antigen retrieval was performed by placing the slides in citrate buffer (0.01 mol/L, pH 6.0) and heating them for 20 minutes using a microwave oven. This was followed by incubation with 3% H_2_O_2_ for 5 minutes to quench the endogenous peroxidase activity. The slides were then incubated with an affinity-purified rabbit polyclonal antibody against human EZH2 (Cell signalling technology, #5246, 1:100) diluted in a 1% BSA-PBST solution (1× PBS with 0.05% Tween-20) for 8 hours at 4 °C. They were then incubated with 100 μL horse-radish peroxidase-conjugated goat anti-rabbit secondary antibody for 30 minutes at room temperature. Sections were counterstained in haematoxylin, dehydrated, cleared, and cover-slipped. The negative control normal and tumour sections were treated in the same manner as all other sections, except that 1.5% normal goat serum was used in place of the primary antibody.

### Scoring of immunostaining

The scoring of the EZH2 immunostaining was based on the intensity and percentage of the positive nuclear staining reference to a previously validated scoring system for EZH2 expression. This system scores the extent of nuclear EZH2 protein staining as follows: 1 (<25% staining of tumour cells), 2 (25–50% staining of tumour cells), 3 (50–75% staining of tumour cells), or 4 (>75% staining of tumour cells). Moreover, the staining intensity was quantified as 0 (indicated no expression), 1 (indicated weak expression), 2 (indicated intermediate expression), or 3 (indicated strong expression). The intensity was multiplied by the extension values to obtain the final immunostaining score (range 0–12), and then the samples were grouped as follows: 1 (score 0), 2 (score 1–2), 3 (score 3–4), 4 (score 6–8), and 5 (score 9–12). In addition, we considered EZH2 expression as a binary variable using a previously described approach for statistical purposes^43^. Group 4 and 5 (Fig. 1d,e) were considered high expression, while the rest (Fig. 1a–c) were considered low expression. In the present study, all sections were scored under high-power magnification, and a minimum of 500 cells in each of the tumours and nontumorous urothelium were included in the calculations. The staining assessment was evaluated by two independent researchers who were blind to the clinicopathological characteristics of the tumours, with a consensus reached in all cases.

### Statistical analysis

Statistical analyses and the generation of the accompanying diagrams were carried out by SPSS 19.0 (SPSS Inc. Chicago, IL, USA). Nonparametric methods, including the Wilcoxon signed rank test, Mann-Whitney test and Kruskal-Wallis test, were used for clinicopathologic variables as appropriate. To detail the differences in cancer-specific survival (CSS) and overall survival (OS) as they related to clinicopathologic parameters and EZH2 protein expression status, Kaplan-Meier analyses were adopted and compared with the log-rank test. To further evaluate the predictive value of clinicopathological categorical variables and the expression levels of EZH2 protein for prognosis of UC patients, univariate and multivariate Cox proportional hazard models were performed. Meanwhile, in order to make sure the robustness of the statistical conclusions, we conduct the resampling test using Bootstrapping method. All P-values are double tailed, and those <0.05 were considered statistically significant.

### Ethics approval and consent to participate

The postoperative prognostic and clinicopathological data were retrospectively analysed in accordance with a protocol approved by the Southwest Hospital, The Third Military Medical University Institutional Review Board (No. KY201403).

## Electronic supplementary material

S1. Bootstrapping

## Data Availability

The analysed data sets generated during the study can be reasonably requested from the corresponding authors.
