# Supplementary material for: Increased expression of EZH2 indicates aggressive potential of urothelial carcinoma of the bladder in a Chinese population
Source: Sci Rep. 2018 Dec 12;8:17792. doi: 10.1038/s41598-018-36164-y (PMC6290761; doi:10.1038/s41598-018-36164-y)
Supplement: Supplementary file 1 — S1. Bootstrapping [file 41598_2018_36164_MOESM1_ESM.rtf]

Increased expression of EZH2 indicates aggressive potential of urothelial carcinoma of the bladder in a Chinese population 
Xiaozhou Zhou1, Nan Liu2, Jingqi Zhang1, Huixiang Ji1, Yuting Liu3, Jin Yang3*, Zhiwen Chen1*
1Urology Institute of People Liberation Army, Southwest Hospital, Third Military Medical University (Army Medical University), Chongqing, China.
2Chongqing University Cancer Hospital & Chongqing Cancer Institute & Chongqing Cancer Hospital 
3 Department of Cell Biology, The Third Military Medical University (Army Medical University), Chongqing, China.
Correspondence: Zhiwen Chen M.D, Urology Institute of People Liberation Army, Southwest Hospital, Third Military Medical University (Army Medical University), Gao Tanyan street 29#, Sha Pingba, Chongqing, China. 400038; E-mail: zhiwen@tmmu.edu.cn
Jin Yang Pro. (jinyang@tmmu.edu.cn), Department of Cell Biology, The Third Military Medical University (Army Medical University), Gao Tanyan street 30#, Sha Pingba, Chongqing, China. 400038; E-mail: jinyang@tmmu.edu.cn
Zhiwen Chen & Jin Yang contributed equally to this work.


Bootstrapping resampling tests (SPSS 19.0 (SPSS Inc. Chicago, IL, USA))

1.	Compared with patients with low expression of EZH2, the hazard ratios of overall death for those with high EZH2 expression was 2.15 (range 1.01-4.56) (p=0.047) in the univariate models. Bootstrapping resampling tests showed robust conclusions.

Variables in the Equation	
	B	SE	Wald	df	Sig.	Exp(B)	
EZH2_PN	.764	.384	3.960	1	.047	2.148	

Bootstrap for Variables in the Equation	
	B	Bootstrapa	
		Bias	Std. Error	Sig. (2-tailed)	95% Confidence Interval	
					Lower	Upper	
EZH2_PN	.764	.008	.396	.038	.060	1.595	
a. Unless otherwise noted, bootstrap results are based on 1000 bootstrap samples
	
Covariate Means	
	Mean	
EZH2_PN	.811	

2.	Compared with patients with low expression of EZH2, the hazard ratios of cancer-related death for those with high EZH2 expression was 3.51 (range 1.08-11.38) (p=0.037) in the univariate models. Bootstrapping resampling tests showed robust conclusions.

Variables in the Equation	
	B	SE	Wald	df	Sig.	Exp(B)	
EZH2_PN	1.255	.601	4.365	1	.037	3.507	

Bootstrap for Variables in the Equation	
	B	Bootstrapa	
		Bias	Std. Error	Sig. (2-tailed)	95% Confidence Interval	
					Lower	Upper	
EZH2_PN	1.255	.163	.676	.024	.341	3.321	
a. Unless otherwise noted, bootstrap results are based on 1000 bootstrap samples
	
Covariate Means	
	Mean	
EZH2_PN	.823	
3.	In the multivariate model, no significant association was observed between EZH2 protein expression and OS (p=0.577). Lymph node invasion maintained its predictive value in overall death (HR=3.28, CI 95% 1.60-6.71, p=0.001). The Bootstrapping resampling tests showed robust conclusions


Variables in the Equation	
	B	SE	Wald	df	Sig.	Exp(B)	
EZH2_PN	.239	.429	.311	1	.577	1.270	
Inv	.385	.420	.842	1	.359	1.470	
G	.454	.336	1.827	1	.176	1.574	
N	1.188	.365	10.574	1	.001	3.280	


Bootstrap for Variables in the Equation	
	B	Bootstrapa	
		Bias	Std. Error	Sig. (2-tailed)	95% Confidence Interval	
					Lower	Upper	
EZH2_PN	.239	.024	.458	.570	-.534	1.299	
Inv	.385	.035	.451	.345	-.381	1.372	
G	.454	.027	.400	.244	-.310	1.242	
N	1.188	.032	.452	.004	.361	2.135	
a. Unless otherwise noted, bootstrap results are based on 1000 bootstrap samples
	
Covariate Means	
	Mean	
EZH2_PN	.823	
Inv	.788	
G	1.248	
N	.159	


4.	In the multivariate model, no significant association was observed between EZH2 protein expression and CSS (p=0.201). Lymph node invasion maintained its predictive value in both cancer-related death (HR=3.23, CI 95% 1.43-7.32, p=0.005) The Bootstrapping resampling tests showed robust conclusions


Variables in the Equation	
	B	SE	Wald	df	Sig.	Exp(B)	
EZH2_PN	.956	.747	1.636	1	.201	2.601	
Inv	.245	.504	.237	1	.626	1.278	
G	.650	.387	2.830	1	.093	1.916	
N	1.172	.417	7.890	1	.005	3.229	


Bootstrap for Variables in the Equation	
	B	Bootstrapa	
		Bias	Std. Error	Sig. (2-tailed)	95% Confidence Interval	
					Lower	Upper	
EZH2_PN	.956	1.383	3.929	.104	-.194	13.227	
Inv	.245	.135	1.112	.626	-.700	1.705	
G	.650	.020	.473	.149	-.283	1.587	
N	1.172	.039	.505	.009	.250	2.179	
a. Unless otherwise noted, bootstrap results are based on 1000 bootstrap samples
	

Covariate Means	
	Mean	
EZH2_PN	.836	
Inv	.782	
G	1.255	
N	.155	
